# Supplementary material for: Radiomics Prediction of Muscle Invasion in Bladder Cancer Using Semi-Automatic Lesion Segmentation of MRI Compared with Manual Segmentation
Source: Bioengineering (Basel). 2023 Nov 25;10(12):1355. doi: 10.3390/bioengineering10121355 (PMC10740947; doi:10.3390/bioengineering10121355)
Supplement: Supplementary file 1 [file bioengineering-10-01355-s001.zip › bioengineering-2639654-supplementary.pdf]

Supplementary materials

S1. Feature extraction

For each VOI, a "Laplacian of Gaussian" (LoG) filter with three sigma values (1.0, 3.0 and 5.0) and a "Wavelet" filter with eight different filtering patterns (high-pass [H] and low-pass [L] filters combined in x, y and z axes, resulting in HHH, HHL, HLH, HLL, LHH, LHL, LLH, and LLL filters) was applied to the original image (12 images in total). For each image, 18 first-order features and 75 texture features were calculated, resulting in (18+75) × 12=1116 features. Additionally, 14 shape features of the three-dimensional VOI were extracted from the original images. Therefore, a total of 1116+14=1130 radiomics features were available for each VOI.

Additionally, the extracted texture features included 24 Gray Level Co-occurrence Matrix (GLCM) features (e.g., Correlation or Contrast), 16 Gray Level Size Zone Matrix (GLSZM) features (e.g., Small Area Emphasis or Zone Variance), 16 Gray Level Run Length Matrix (GLRLM) features (e.g., Gray Level Variance or Gray Level Non-Uniformity), 14 Gray Level Dependence Matrix (GLDM) features (e.g., Dependence Non-Uniformity or High Gray Level Emphasis), and 5 Neighborhood Gray Tone Difference Matrix (NGTDM) features (e.g., Complexity or Strength).

S2. The feature selection result of the model based on manual segmentation

Among the total of 1130 radiomics features extracted from each VOI, 24 features with non-zero coefficients (Table S2) were finally retained for inputting into the radiomics model according to the least absolute shrinkage and selection operator

(LASSO) algorithm with an optimal lambda (λ) value of 0.024 ($\ln \lambda = -3.730$; Figure S1).

Table S1: T2-weighted imaging scanning acquisition protocols

Parameters	Center 1 (The Tenth Affiliated Hospital of Southern Medical University (Dongguan People's Hospital))	Center 2 (Sun Yat-Sen University Cancer Center)
Scanner	MAGNETOM Skyra, Siemens, Germany	UMR 780, United Imaging Healthcare, Shanghai, China
TR (ms)	7500	4000
TE (ms)	101	120
FOV (cm)	20	20
Matrix	320*320	336*269
Slice thickness (mm)	4	3
Slice gap (mm)	0.4	0.6
Number of excitations	2	1.5

Note: TR = repetition time; TE = echo time; FOV = field of view.

Table S2: The selected radiomics features of the model based on manual segmentation for classifying NMIBC and MIBC

Feature name	Coefficient
original_firstorder_Kurtosis	0.136
original_shape_MinorAxisLength	0.041
wavelet-LLL_firstorder_Skewness	0.038
log-sigma-1-0-mm-3D_firstorder_Skewness	0.036
wavelet-HLL_firstorder_Skewness	0.034
log-sigma-3-0-mm-3D_glszm_GrayLevelNonUniformity	0.032
wavelet-HHL_gldm_LargeDependenceLowGrayLevelEmphasis	0.029
log-sigma-1-0-mm-3D_glszm_ZoneEntropy	0.029
original_shape_Sphericity	-0.025
original_glcm_Correlation	0.018
original_glcm_MCC	0.017
log-sigma-1-0-mm-3D_glrlm_RunVariance	0.014
wavelet-HHL_firstorder_Mean	0.013
log-sigma-5-0-mm-3D_firstorder_Median	0.011
wavelet-LHL_glcm_MCC	-0.009
log-sigma-1-0-mm-3D_glcm_ClusterShade	0.009
log-sigma-5-0-mm-3D_glcm_InverseVariance	0.008
log-sigma-5-0-mm-3D_gldm_LargeDependenceLowGrayLevelEmphasis	-0.007
wavelet-HLL_glszm_SmallAreaEmphasis	-0.007
wavelet-HLH_glszm_GrayLevelNonUniformity	0.007
log-sigma-3-0-mm-3D_glszm_SmallAreaEmphasis	0.006
wavelet-LHL_ngtdm_Complexity	-0.004
wavelet-HHL_glrlm_LongRunLowGrayLevelEmphasis	0.004
wavelet-HHL_glcm_MCC	-0.002

Note: Each feature was named by concatenating the image type from which the feature was extracted, feature group, and feature name using underscores. For example, original_firstorder_Kurtosis was a feature extracted from the original image, first-order group, with feature name Kurtosis. All these features are texture features.

Glcm, gray level co-occurrence matrix; glszm, gray level size zone matrix; gldm, gray level dependence matrix; glrlm, gray level run length matrix; ngtdm, neighbourhood gray tone difference matrix.

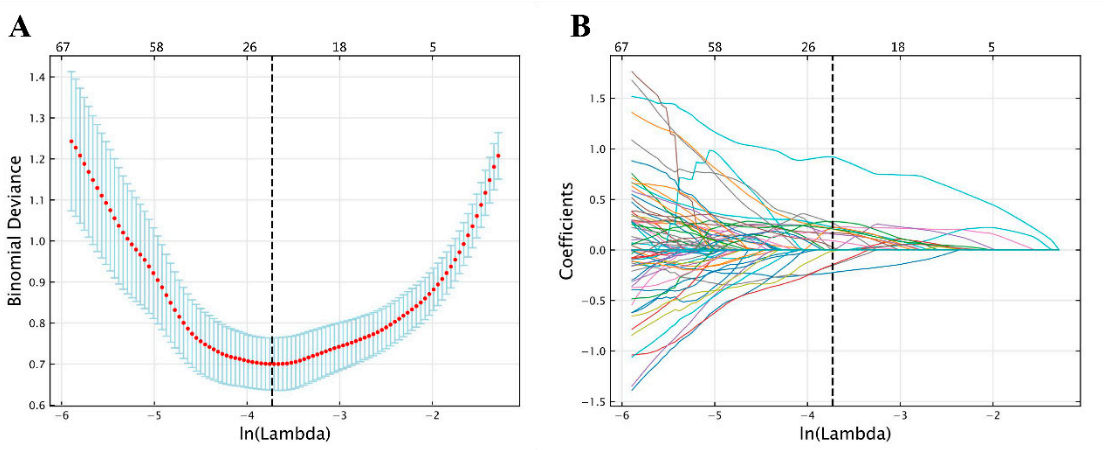

Figure S1: Feature selection results following LASSO based on features extracted from manual segmentation results. (A) The selection of the LASSO model's tuning parameter lambda (λ), based on 10-fold cross-validation using minimum criteria. The vertical dashed line indicates the optimal value of λ. (B) The coefficient profile plot. The vertical dashed line represents 24 radiomics features with non-zero coefficients selected with optimal λ value. In each plot, the x-axis at the bottom shows ln(λ), while the x-axis at the top shows the number of remaining radiomics features that vary with λ. LASSO, least absolute shrinkage and selection operator.
